# Plasmodesmata and their role in the regulation of phloem unloading during fruit development

**DOI:** 10.1016/j.pbi.2021.102145

**Published:** 2021-12

**Authors:** Candelas Paniagua, Besiana Sinanaj, Yoselin Benitez-Alfonso

**Affiliations:** Centre for Plant Sciences, School of Biology, University of Leeds, Leeds, LS2 9JT, UK

**Keywords:** Phloem unloading, Fruit development, Plasmodesmata, Symplasmic transport, Apoplastic transport

## Abstract

Fruit consumption is fundamental to a balanced diet. The contemporary challenge of maintaining a steady food supply to meet the demands of a growing population is driving the development of strategies to improve the production and nutritional quality of fruit. Plasmodesmata, the structures that mediate symplasmic transport between plant cells, play an important role in phloem unloading and distribution of sugars and signalling molecules into developing organs. Targeted modifications to the structures and functioning of plasmodesmata have the potential to improve fruit development; however, knowledge on the mechanisms underpinning plasmodesmata regulation in this context is scarce. In this review, we have compiled current knowledge on plasmodesmata and their structural characterisation during the development of fruit organs. We discuss key questions on phloem unloading, including the pathway shift from symplasmic to apoplastic that takes place during the onset of ripening as potential targets for improving fruit quality.

## Highlights

• Plasmodesmata are structures that mediate symplasmic transport between plant cells.
• Symplasmic cell-to-cell transport regulates phloem unloading and organ development.
• Symplasmic phloem unloading of assimilates and signalling molecules regulate fruit growth.
• Targeted modifications in plasmodesmata could be used to improve fruit development.

## Introduction

Fruits are a fundamental part of a healthy diet. The World Health Organization (WHO) recommends a minimum daily intake of 5 portions or 400 g of fruit and vegetables each day to reduce the risk of non-communicable diseases, such as cardiovascular diseases, osteoporosis, and certain types of cancers ([1] and references therein). The demand for fruits and vegetables will increase as current estimates indicate that the global population will increase to ∼9 billion by the middle of this century [2]. Agricultural improvements to enhance the yield and quality of these products will have a positive impact on human health and the economy. Understanding the processes and molecular mechanisms that influence fruit development is a crucial step towards implementing strategies to secure their future availability and nutritional value.

Carbon partitioning between source organs (e.g. photosynthetically active leaves) and dividing/growing sink tissues, coupled with the transport and metabolism of resources within receiving organs, are important determinants in fruit initiation, growth and organoleptic properties [3]. Photoassimilates, hormones, certain polypeptides and RNAs translocate through the phloem and are unloaded via either the apoplastic or the symplasmic pathway to control the development of new organs [4, 5, 6, 7, 8]. The apoplastic pathway involves export and import through membrane-localised transporters such as the Sugars Will Eventually be Exported Transporters (SWEETs), hexose transporters (HTs) and the sucrose transporters (SUTs), whereas the symplasmic pathway is mediated by cytoplasm-to-cytoplasm transport via plasmodesmata (PD) [9, 10, 11].

PD are small intercellular channels (or pores) traversing cell walls to connect the cytoplasm of neighbouring cells [12]. Many examples in the literature point to a role for PD in phloem loading and unloading to facilitate sugar partitioning and long-distance signalling (see the study by Yan and Liu [9] for a recent review). These processes regulate the development, cell fate and differentiation of sink tissues, including meristems, and the formation of new organs [12]. Modifying PD structure in the phloem pole pericycle (PPP)-endodermis interface restricts root meristem growth in *Arabidopsis thaliana* [13]. The accumulation of soluble sugars and starch in developing leaves of the maize mutant *carbohydrate partitioning defective 33* (*cpd33*) was tightly linked to defective symplasmic transport into sieve elements [14]. Phloem loading, transport and unloading are also essential to coordinate fruit development in response to changes in a plant's physiological state and the environment [11,15], but the role of PD in this context is not yet fully understood [16].

A true fruit initiates with the fertilisation of ovules in flowers followed by rapid cell division and expansion of ovary tissues ([17] and references therein). In fleshy fruits, cell expansion and increase in water content occur at a dramatic rate just before the onset of ripening. During ripening, changes in sugars, anthocyanins and other compounds accompany modifications in cell wall properties and cell mechanics that regulate fruit quality and shelf life [17]. Multiple hormonal pathways control fruit development [18]. Auxin levels increase during fertilisation, while gibberellic acid stimulates fruit growth. Before the onset of ripening, auxin declines while abscisic acid (ABA) (in non-climacteric fruits) and ethylene (in climacteric fruits) are reported to increase. Changes in hormonal balances lead to significant modifications in the fruit transcriptome and metabolome [18,19]. Auxin responsive factors activate basic helix-loop-helix transcription factors during fertilisation [20,21], which also contribute to ripening [22]. MADS-box genes are induced at later stages to control fruit size and shape by modulating the activity of cell wall enzymes, among other components [19,23,24].

Few (mainly structural) studies evidence the role of PD in fruit development [16]. A compelling publication compares wild (sour) and cultivated Chinese jujube [25]. The authors found high PD density at the phloem interface in the cultivated sweet variety while, in the same interface, the wild sour cultivar had few or no PD. The results suggest that symplasmic phloem unloading is important for the accumulation of soluble sugars in the cultivated fruit. This one example highlights the underestimated value of targeting PD as a route for fruit improvement.

In this review, we discuss new findings on the mechanisms regulating PD permeability and their implications in our understanding of processes that regulate fruit development. By evaluating this information, new research avenues are proposed for knowledge gathering, aiming to find a path for the exploitation of PD as a future target for the improvement of fruit crops.

## An overview of PD structure and regulation

**Figure 1:**
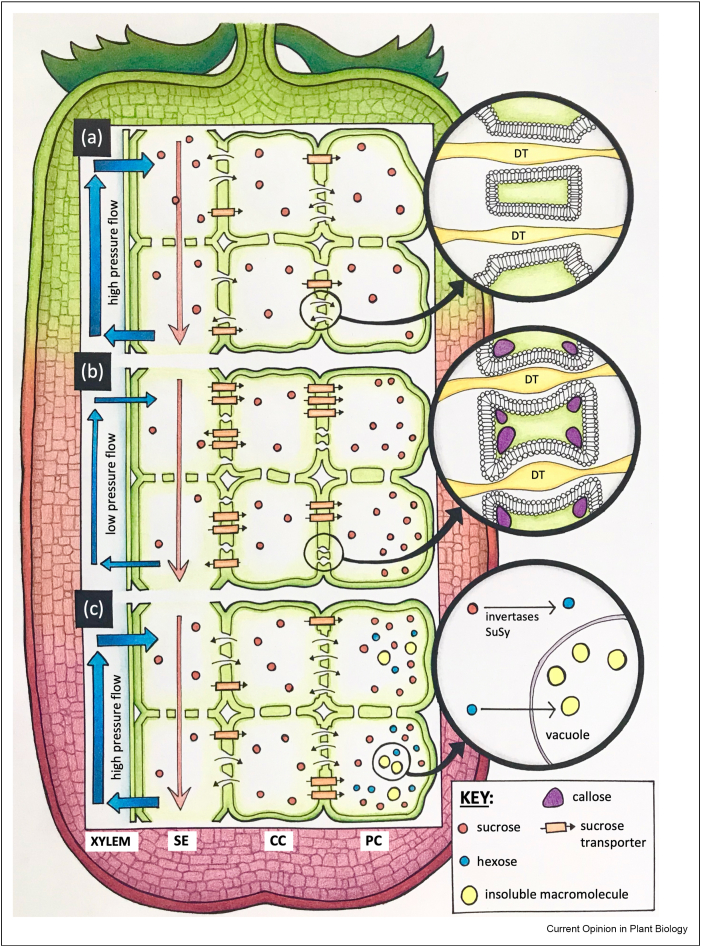
**Schematic representation of the phloem unloading and transport pathways during fruit development.** From left to right, the xylem, sieve element (SE), companion cell (CC) and parenchymal cell (PC) within fruit are represented. **(a)** Symplasmic unloading in fruits, such as tomato, is driven by high-pressure flow and low sugar (e.g. sucrose) content in recipient cells and open plasmodesmata (PD) connections, which may be present as ‘pit fields’. Apoplastic transporters contribute to sugar transport to a lesser extent. Open PD are depicted in the panel detail, displaying the phospholipid bilayer membrane and traversing desmotubule (DT) embedded in cell walls. **(b)** During maturation and ripening, phloem unloading in some fruits shifts to the apoplastic pathway accompanied by the up-regulation of genes encoding for sucrose transporters and the deposition of callose at PD cell walls, as shown in the panel detail. Because of the resolution of this hand-drawn figure, sucrose exporters and importers (e.g. SUTs and SWEETs) are represented collectively by the sucrose transporter symbols, with arrows indicating the direction of transport (in and out of the cell). **(c)** Some fruits are able to maintain the symplasmic pathway open at the later stages of development by increasing phloem pressure (e.g. as a result of daytime transpiration losses) or changing their sucrose metabolic rate and adopting compartmentation strategies (i.e. enhanced conversion of sucrose in hexoses by the action of SuSy and invertases and recruitment in vacuoles in the form of insoluble molecules), as shown in the panel detail.

PD structure is studied using electron microscopy and/or tomography [26, 27, 28]. PD can be described as plasma membrane (PM) lined pores containing a central tubular structure of appressed endoplasmic reticulum (ER) named the desmotubule (DT). A simplified representation of PD is shown in Figure 1. Proteins, that is, synaptotagmins or membrane-bound C2 domain proteins (MCTPs), tether the PM and DT membranes, providing stability to these structures [26]. CPD33 encodes an MCTP protein, which likely explains its role in regulating PD transport and sugar unloading, as discussed in the introduction [14]. The space between the DT and the PM, called the cytoplasmic sleeve, is traditionally thought to be the main route for molecular transport [27]. A recent study in meristem and cell culture argues with this theory reporting a high proportion of PD that appeared to lack cytoplasmic sleeves (named occluded or type I) despite the high molecular permeability of these tissues [28]. Cell walls around PD are also different. The presence of callose, a cell wall polysaccharide detectable using immuno-gold or immuno-fluorescent microscopy or using aniline blue staining, is deemed responsible for changes at PD [29].

PD originate during cytokinesis and undergo structural modifications during cell expansion and differentiation [30]. Secondary PD can be formed post-cytokinesis through a yet-to-be-determined mechanism. Complex structures (e.g. twinned or branched channels) are found in matured tissues and are traditionally associated with a reduction in the size of the molecules that can be transported via PD (or size exclusion limit) [30]. Multi-chambered clusters of PD, called pit fields, are described in some organs, such as developing fruits [27,30] (consult the next section).

Specialised PD structures have also been described in phloem cells. In root tips, ‘funnel-PD’ connect protophloem sieve tubes and PPP cells [31]. Funnel-shaped PD are wider on the phloem sieve elements (SEs) side and narrower towards the PPP cells. Modelling indicates that this structural configuration facilitates solute unloading even when the osmotic gradient is low [31].

Aside from their physical ultrastructure, the molecular composition of PD regulates molecular transport [27]. Proteomic approaches identified cell wall metabolic enzymes and several signalling proteins attached to PD [32,33]. Lipidomic profiles of PD membranes revealed that these microdomains are rich in sterols and sphingolipids with saturated very long-chain fatty acids [13]. Interestingly, modifying lipid composition, using chemicals or mutations in specific sphingolipid long-chain base 8 desaturases (*Arabidopsis sld1sld2*) or the gene *Phloem unLoading Modulator* (*PLM*), alters symplasmic permeability [13,34,35]. Mutations in *PLM* change the frequency of type I PD in the PPP-endodermis interface, linking PD structures and membranes composition [13].

Cell walls surrounding PD are enriched in the beta-1,3 glucan callose, but other components, such as pectins, may also play a role in the regulation of these structures (see the study by Amsbury et al. [36] for a recent review). Cell walls physically constrict the channel aperture, affecting molecular flux (Figure1b inset). Recent findings suggest that callose might interact with other cell wall components, including cellulose, to fine-tune the elasticity, plasticity and ductility of domains surrounding PD [37]. Specific family members of the glucan synthase-like (GSL, i.e. callose synthases) and beta-1,3-glucanases (BGs) are responsible for callose metabolism at PD [38]. Other proteins such as the PD Callose Binding domains (PDCBs) and PD Located Proteins (PDLPs) regulate callose accumulation [38]. Computational modelling has identified cell wall length/thickness as a major contributor to changes in symplasmic permeability, bringing a new perspective to the role of cell walls in PD communication [39].

## PD structure and composition in fruits

Electron-micrographs showed the presence of branched and unbranched PD in the mesocarp of avocado fruits [40]. In tomatoes, pit fields of ∼1–5 μm in diameter have been observed in the pericarp [29]. Scanning electron microscopy showed circular depressions with ridge-like features of 80–140 nm in width spaced at 300–350 nm [29]. Fluorescent imaging also revealed presumed PD connections in other fruits such as bananas and mangoes [41]. Calcofluor-white staining of cellulose in mature fruits revealed regions between parenchyma cells (PCs) that resemble pit fields in these fruits [41]. It is not clear how pit fields form or what their functional significance is, however, recent modelling work suggests that for the same PD density, pit fields clustering may decrease effective permeability. The model also revealed that, in thick cell walls, the effect of clustering on permeability is less strong [39].

In the context of fruit development, there are very few studies on PD composition. Aside from being rich in callose and depleted in cellulose, pit fields in apple and tomato pericarp appear acidic with an abundance of homogalacturonan (HG), a pectic polysaccharide of a linear alpha-1,4-linked galacturonic acid (GalA) backbone [29,36,42]. Immuno-probes also showed rhamnogalacturonan I (RG I, pectin formed by GalA and rhamnose subunits) with (1–5)-α-L-arabinan side chains enriched at pit field regions [29,36]. The biological significance of pectins found at pit fields is yet to be uncovered, but potential interactions with callose may be critical for intercellular permeability and the physico-mechanical properties of the cell wall.

**Figure 2:**
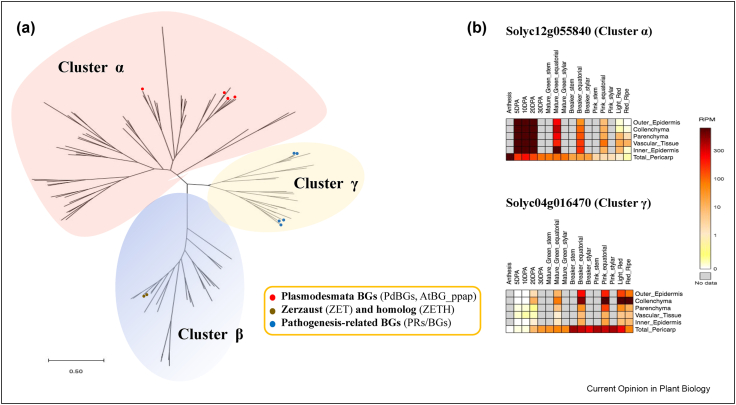
**Phylogenetic relations and expression of cell wall β-1,3-glucanases (BGs) in tomato fruit. (a)** The overall structure of a phylogenetic tree represents relationships of BG genes in tomato and Arabidopsis. Amino acid sequence alignment and tree were created using MEGA-X40 (Maximum Likelihood method and JTT matrix-based model). The tree is at scale, with branch lengths measured in the number of substitutions per site. Three clades (α, β and γ) are identified as previously described for *Arabidopsis thaliana* [46]. Coloured spots indicate the position of known Arabidopsis genes (see legend). **(b)** The expression of two tomato BG representatives located in cluster α and γ was extracted from the SGN Tomato Expression Atlas (https://tea.solgenomics.net/). The heat map indicates expression in reads per million (RPM) in the different tissue types and at different developmental stages. DAA: days after anthesis. The full tree and expression analysis is reported in Paniagua et al., 2021 [45].

Several publications confirm the presence of GSL and BG enzymes in fruit cell walls [42, 43, 44]. In tomato fruits, a decline in GSL enzymatic activity during ripening was reported [43], while there was high expression levels of ethylene-induced BGs during the early ripening stages in banana fruits [44]. We have used a combination of phylogenetic and transcriptomic tools to identify cell wall β-1,3-glucanases (BG) expressed during the development of tomato fruits (Figure 2) [45]. Tomato BG genes are organised into three phylogenetic clusters (α, β and γ), as previously described for *A. thaliana* [46]. Through homology with Arabidopsis, we propose that cluster α comprises candidate PD enzymes (Figure 2a). Interestingly, the expression of candidate orthologs to Arabidopsis PdBG proteins (such as Solyc12g055840) decreases during fruit maturation and ripening in contrast to Solyc04g016470, a BG belonging to cluster γ, is up-regulated (Figure 2b) [45]. Experiments are in progress to confirm the localisation and function of these BGs.

## Phloem unloading pathways in developing fruits: a role for PD

Phloem unloading into sink organs is driven by osmotic pressure differences, sugar metabolism and compartmentation strategies (Figure 1). Sucrose is the main phloem-translocated sugar, hydrolysed to hexose by invertases or sucrose synthases (SuSy) in post-phloem cells and stored in the form of insoluble macromolecules in the vacuole [3,11,47,48]. The Rosaceae family, which includes apples, transport sucrose and sugar alcohols, such as sorbitol and mannitol via the phloem, until they reach sink PC, where they are converted into fructose and glucose by sorbitol dehydrogenase and mannitol dehydrogenase, respectively [49]. Fruits in the Cucurbitaceae and Scrophulariaceae families transport raffinose oligosaccharides (RFO) which enter the SE to be unloaded into the fruit tissues [50]. RFO is metabolised into sucrose and galactose by α-Galactosidases (α-Gal) at their destination and subsequently hydrolysed into hexoses by invertases and/or SuSy [48].

In developing fruits, an osmotic pressure gradient (Munch pressure flow) is created by differences in sugar accumulation between source and developing organs, driving the phloem unloading of sugars from the SE to companion cells (CCs) and into the PC [11]. To maintain phloem osmotic pressure and photoassimilate unloading, water is exported from the phloem synchronously by diffusion or via apoplast transporters, such as aquaporins [51,52]. The presence of PD in the SE-CC connection has been reported as evidence of symplasmic unloading in tomatoes [53], kiwi [54], grape berries [55] and jujube [25] (Table 1). In other fruits, such as apples [49], strawberries [56], cucumbers [57, 58, 59] and watermelons [60], apoplastic phloem delivery predominates at all developmental stages (Table 1). Phloem unloading can switch from the symplasmic to the apoplastic pathway during fruit development according to physiological demands and environmental conditions [11,48] (Figure 1). In tomatoes, phloem unloading switches from the apoplastic to the symplasmic pathway in ovary walls and during fruit initiation stages (∼12–15 days after anthesis, DAA) [53]. Studies using symplasmic and apoplastic tracers and structural determinations indicate a separate transition from symplasmic to apoplastic phloem unloading at the onset of ripening (23–25 DAA) [61]. This transition correlates with a reduction in the source-to-sink osmotic gradient because of the accumulation of soluble sugars at the later stages of fruit development [55]. The increased osmotic potential in the developing grape berry is likely responsible for the switch to apoplasmic unloading, decoupling the storage PC from the symplasm of the conducting phloem [55]. Changes in sink metabolism, compartmentation of soluble sugars and loss of water via transpiration allow some fruits to use the symplasmic pathway during all ripening stages (Figure 1c). These strategies keep osmotic pressure low by converting the sugars into a less osmotically active form such as starch ([62] and references therein). In Japanese plums, the symplasmic and apoplastic pathways appear to co-exist and be used alternatively throughout the day according to changes in environmental conditions [62]. Water loss during the day via transpiration reduces turgor potential, which favours symplasmic unloading into the fruit [62].

**Table 1:** Summary of information on phloem unloading pathways associated with fruit development based on structural and functional studies.

Fruit	Main phloem unloading route	Mobile sugar	Experimental evidence	Reference
Grape (*Vitis vinifera* x *labrusca*)	Symplasmic at early stages. Apoplastic later in development.	Sucrose	Structural studies show numerous PD at SE–CC complex. Dense deposits and diffusion of CF and viral MP indicate PD blocked at late stages. Increase in apoplastic sugars and acid invertase expression at the onset of ripening.	[55,66]
Strawberry (*Fragaria* x *ananassa*)	Apoplastic pathway.	Sucrose Sorbitol	The abundance of PD between PCs, rare presence between SE-CC complexes. Transport of the symplasmic tracer CF is restricted.	[56]
Watermelon fruit (*Citrullus lanatus*)	Apoplastic pathway.	Sucrose	CF does not diffuse out of the phloem into the fruit.	[60]
Chinese jujube (*Zizyphus jujuba*)	Apoplastic transport at early and late stages. Symplasmic transport at middle stage.	Sucrose	Structural studies and symplasmic tracers show connections at SE–CC complex during the middle stage but not during early and late developmental stages. Cultivar variations: PD observed at early stages in cultivated jujube but not in wild sour jujube.	[25]
Walnut (*Juglans regia)*	Apoplastic pathway in fruit fleshy pericarp. Symplasmic pathway in the seed pericarp.	Sucrose	The high density of PD in SE-CC and PCs in seed pericarp but low density in the fruit fleshy pericarp. CF is restricted in the fleshy pericarp but moves into the seed pericarp.	[63]
Kiwi *(Actinidia deliciosa cv. Qinmei)*	Apoplastic pathway.	Sucrose	PD were observed in CC-SE, but low density or none were observed between SE and PC. Restricted diffusion of CF at all developmental stages. Note: early work, in a different cultivar, symplasmic phloem unloading was suggested during fruit development.	[54]
Apple (*Malus* x *domestica*)	Apoplastic pathway.	Mannitol Sorbitol Sucrose	PD are rarely observed between SE-CC complexes and PCs. None were observed in the major bundle at the end of development. Tracer studies confirm that the phloem is symplasmically isolated.	[49]
Cucumber (*Cucumis sativus*)	Apoplastic pathway.	RFOs stachyose	Structural studies show PD are rarely observed between SE-CC complexes and PCs. Symplasmic tracer showed phloem is symplasmic isolated during development.	[57,58]
Tomato (*Solanum lycopersicum*)	Symplasmic early during fruit initiation. Transition to apoplastic transport at ∼23 DAA.	Sucrose	Symplasmic and apoplastic tracers and [^14^C]-feeding used to address unloading. Symplasmic unloading is restricted before anthesis but establishes 2 DAA and early during fruit initiation. The apoplastic pathway operates in ovaries and later in fruit development.	[53,61]
Japanese plum (*Prunus salicina* L.) cv. Angeleno	Symplasmic and apoplastic pathways coexist.	Sorbitol Sucrose	Vascular flow, skin transpiration and pressure potentials were used to predict the unloading pathway.	[62]

Abbreviations: PD= Plasmodesmata; SE= Sieve Elements; CC= Companion Cells; PCs= Parenchyma cells; CF= Carboxyfluorescein; MP = movement protein; RFOs = Raffinose Family Oligosaccharides; DAA = days after anthesis.

Different tissue types or cultivars might also differ in their phloem unloading strategies. In walnut fruits, unloading in the seed pericarp is mainly symplasmic, while in the fleshy pericarp, it is apoplastic [63]. In a study comparing wild (sour) and cultivated Chinese jujube, detailed transcriptome analysis, ultrastructure observations and measurement of soluble sugar indicate that PD density and sugar accumulation correlate [25]. In cultivated jujube, PD density at the SE-CC interface increased during the white, mature stages of fruit development, while the wild cultivar had few or no PD during these developmental stages. The expression of sugar transporters was higher in cultivated jujube than wild jujube; thus, the apoplastic pathway also contributes to the differences in sugar accumulation [25].

PD ultrastructure and function reflect the shifts in phloem unloading mechanisms [16]. The activity of cell wall invertases (which hydrolyse sucrose into hexoses) and sugar transporters (SUTs, HTs and SWEETs) are also coordinated to regulate this shift [11,15]. A recent study in tomatoes identified SlSWEET15 as the major isoform mediating apoplastic sucrose unloading during fruit expansion [64]. Zhang et al. (2006) [55] studied the development of grape berries (*Vitis vinifera*) and found numerous PD connections in the SE–CC complex and the interface of the associated PC. Around 10–20% of this PD appeared occluded (presumably by callose deposits) late in development, and 5% showed branched structures. Concomitantly, a reduction in the transport of the symplasmic reporter carboxyfluorescein (CF) and a *Cucumber mosaic virus* movement protein suggested a reduction in PD conductivity [55]. The expression of cell wall acid invertases and the presence of soluble sugars in the apoplast corroborated the transition to the apoplastic unloading pathway during ripening [55]. On the other hand, ultrastructure studies in apoplastic phloem unloaders, such as apples and strawberries, indicate sparsity or absence of PD in the SE-CC interface with PC during all stages of development [49,56]. In apples, the expression of members of the sugar transporters family (e.g. *SUT2* and *SUT4* genes) facilitate sugar accumulation via the apoplastic pathway [65]. In kiwi fruits, high numbers of PD were observed in the SE-CC interface but not in the post-phloem unloading domains (CC-PC or SE-PC) concomitant with restricted symplasmic unloading of CF [54]. RNA-seq analysis in grapes collected after phloem unloading stops (at the end of ripening), reveals downregulation in the expression of the SWEET transporters, of cell wall genes and aquaporins, marking the cessation of growth [66].

The phloem does not only transport sugars. Phytohormones (e.g., ABA, auxins, cytokinins) and mRNAs have been found in phloem exudates supporting a role in long-distance signalling [7,8]. Proteins involved in defence against pathogens, such as viruses, were also isolated in the phloem sap of mulberry and melon [67]. Other symplasmic mobile molecules delivered via the phloem include transcription factors and miRNAs, with a role in flowering and fruit development. Koenig and Hoffmann-Benning (2020) [6] summarise these mobile factors and their function. A typical example is the Flowering Locus T (FT) and FT-like proteins (FTL) that move from leaves to shoot apical meristems to induce flowering in multiple commercially relevant fruit species such as cucurbits, apples and tomatoes [6]. Interestingly, in cucurbits, where sugar unloading is mainly apoplastic, two spatially separated phloem systems have been described, one mainly dedicated to sugar transport (fascicular phloem) and another presumed to be specific for proteins, peptides and other mobile molecules (extrafascicular phloem) [68]. This suggests that there are mechanisms for independent control of phloem unloading for different mobile molecules. Finally, given the extraordinary importance of hormone signalling in all stages of fruit development, it is relevant to cite recent evidence of a role for callose and PD in the regulation of auxin gradients and gibberellic acid/ABA signalling during bud re-activation after dormancy in poplar [69,70].

## Conclusions and future perspectives

Enhancing the nutritional quality of fruits and vegetables and improving crop yields are long-term goals of horticultural research. Modulating phloem unloading may hold the key to improve the accumulation of sugars and the transport of other molecules that control fruit initiation and development [3,5,16]. A recent publication describes how increasing sink strength and phloem unloading by changing the expression of the vacuolar sugar transporter ClVST1 triggers modifications in sugar accumulation and total biomass in watermelon [60]. In this review, we highlight the role of PD and symplasmic transport in phloem unloading and fruit development.

Finding strategies to modify PD or pit fields has the potential to lead to improvements in fruit sugar content, texture and/or responses to climatic changes and pathogenic attacks [16]. There are knowledge gaps concerning the mechanism for PD structural development and regulation in fruits. A shift from the symplasmic to the apoplastic pathway before ripening was reported in some fruits (Table 1); however, further research is required to determine the specific mechanism that controls this shift and the consequences of delaying or modifying it. Identification of the genes involved in these transitions will be key to implement biotechnological modifications or breeding strategies in fruit crops [71,72]. Integrators of energy, nutrient and hormone signalling, such as the serine/threonine kinase Target of Rapamycin (TOR), might become a potential avenue to modify PD and sugar phloem unloading in fruits [73]. TOR is critical for signalling sugar transport from mature leaves to sink tissues, regulating symplasmic phloem loading/unloading [73].

Regulation of callose levels in fruits might also be a suitable target to modify PD and phloem transport [74, 75, 76]. Guerriero et al. 2014 [74] correlated the expression of callose synthetic enzymes with the concentration of soluble sugars at different stages of apple development. In a non-fruit context, ectopic callose deposition in the phloem in the *Carbohydrate partitioning defective1* (*Cpd1*) mutant in maize lead to defective sucrose export and the accumulation of starch and soluble sugars in leaves [77]. Infection with *Candidatus Liberibacter* in citrus tree induces callose accumulation at PD connecting CC and SE, leading to reduced symplasmic transport and delayed sucrose export in comparison to uninfected leaves [78].

New mathematical models to estimate symplasmic permeability between different cell types during fruit development are required. Recently, computational and visualisation tools have been developed to quantitatively analyse symplasmic trafficking and evaluate the importance of PD in water transport and tissue hydraulic conductance, which are essential factors controlling fruit growth [79]. The assembly of these tools will help to predict the contribution of PD to carbon partitioning, protein and RNA signalling, turgor pressure and cell wall mechanics in fruits.

The role of PD in fruit development is complex, and research in this area is still in its infancy. We hope this review inspires researchers to investigate the hidden potential of modifying these structures as a route to modulate phloem unloading and cell-to-cell transport, with the goal to enhance fruit quality and nutritional value.

## Declaration of competing interest

The authors declare that they have no known competing financial interests or personal relationships that could have appeared to influence the work reported in this paper.
